# A Case of Pylephlebitis Without Intra-abdominal Infection Secondary to Pneumonia Caused by Hypermucoviscous Klebsiella pneumoniae

**DOI:** 10.7759/cureus.69428

**Published:** 2024-09-14

**Authors:** Kohei Yamamoto, Isao Hasegawa, Yoshifumi Suga, Yukari Kano

**Affiliations:** 1 Respiratory Medicine, Japanese Red Cross Kyoto Daiichi Hospital, Kyoto, JPN; 2 Respiratory Medicine, Saiseikai Shiga Hospital, Moriyama, JPN; 3 Respiratory Medicine, Japanese Red Cross Kyoto Daini Hospital, Kyoto, JPN

**Keywords:** hypermucoviscous klebsiella pneumoniae, klebsiella pneumoniae pneumonia, portal vein thrombosis, pylephlebitis, string test

## Abstract

Pylephlebitis is an infective, suppurative thrombosis of the portal vein that is often a complication of intra-abdominal infections. Herein, we report a rare case of hypermucoviscous *Klebsiella pneumoniae* pneumonia complicated by pylephlebitis. The patient was administered antibiotics and anticoagulants. His pneumonia improved; however, the thrombus in the portal vein did not shrink, and the patient ultimately died of liver failure. Furthermore, hypermucoviscous *K. pneumoniae* is involved in the formation of portal vein thrombosis, and if bacteremia persists even after pneumonia has improved, investigating possible complications, including portal vein inflammation, is necessary.

## Introduction

Pylephlebitis is an infective, suppurative thrombosis of the portal vein. It occurs as a complication of intra-abdominal infections such as diverticulitis, appendicitis, cholangitis, and liver abscess [1]. *Klebsiella pneumoniae* is one of the primary bacteria that can cause pylephlebitis. The hypermucoviscous type of *K. pneumoniae* is associated with a high risk of invasive infections such as liver abscess, meningitis, and endophthalmitis [2].

Herein, we report a rare case of pylephlebitis without intra-abdominal infection secondary to pneumonia caused by hypermucoviscous *K. pneumoniae*.

## Case presentation

An 83-year-old male with a medical history of myelodysplastic syndrome (MDS) and Alzheimer's disease visited the emergency department (day 1) because of a fever and dyspnea that had developed one day prior. He was in shock on admission, with respiratory and circulatory failure. His vital signs were blood pressure of 94/60 mmHg, pulse rate of 208 beats per minute, saturation of 96% with 7 L of oxygen, respiratory rate of 34 breaths per minute, and body temperature of 40.2℃. Blood tests revealed elevated aspartate transaminase, alanine transaminase, C-reactive protein (CRP), and bilirubin; acute kidney injury; increased white blood cells; and abnormal coagulation parameters (Table 1). The patient's acute-phase disseminated intravascular coagulation (DIC) score was 8, which indicated the presence of DIC. The electrocardiogram showed a paroxysmal supraventricular tachycardia (PSVT) waveform.

**Table 1:** Laboratory data ALT, alanine transaminase; AST, aspartate transaminase; CRP, C-reactive protein; D-Bil, direct bilirubin; FDP, fibrinogen degradation products; Hb, hemoglobin; Plt, platelets; PT, prothrombin time; PT-INR, prothrombin time-international normalized ratio; T-Bil, total bilirubin

	Day 1	Day 3	Day 6	Day 10	Day 14	Day 20	Day 27	Reference range
Blood chemistry								
AST (U/L)	57	249	141	74	58	63	65	13-30 (U/L)
ALT (U/L)	38	209	142	76	51	53	39	10-42 (U/L)
T-Bil (U/L)	2.31	3.10	5.12	7.06	6.89	7.66	9.60	0.4-1.5 (U/L)
D-Bil (U/L)	1.00	1.54	3.35	4.25	4.15	5.47	7.69	0-0.2 (U/L)
CRP (mg/dL)	20.96	37.95	25.12	12.87	7.15	6.91	14.17	0-0.14 (mg/dL)
Blood count								
Hb (mg/dL)	9.8	8.4	8.2	6.6	5.9	5.8	4.3	13.7-16.8 (mg/dL)
Plt (×10^3^/μL)	72	36	8	25	12	9	6	158-348 (×10^3^/μL)
Coagulation								
PT (%)	48.3	31.9	62	50.1	60.3	44.5	23.9	80-130 (%)
PT-INR	1.68	2.25	1.36	1.64	1.39	1.79	2.88	0.85-1.15
FDP (μg/mL)	42.9	50.4	59.0	44.5	49.3	29.8	24.9	0-5 (μg/mL)

**Figure 1:**
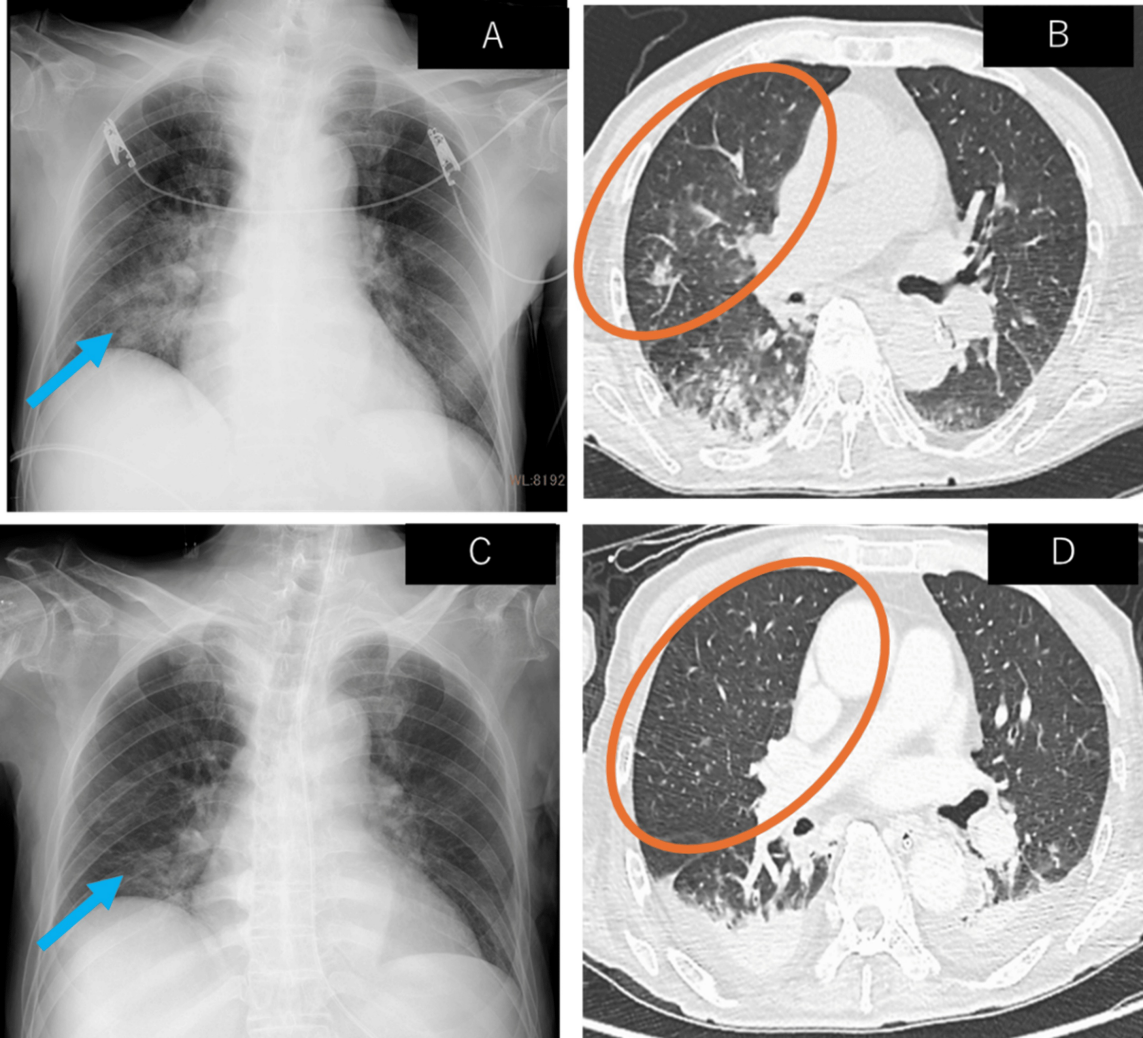
Chest radiography and computed tomography images (A) Radiography showed ground-glass opacities in the right lower lung field on day 1. (B) Computed tomography (CT) showed ground-glass opacities in the right upper and lower lobes on day 1. (C and D) The abnormal shadows observed on radiography and CT scans on day 1 were improved by day 6

**Figure 2:**
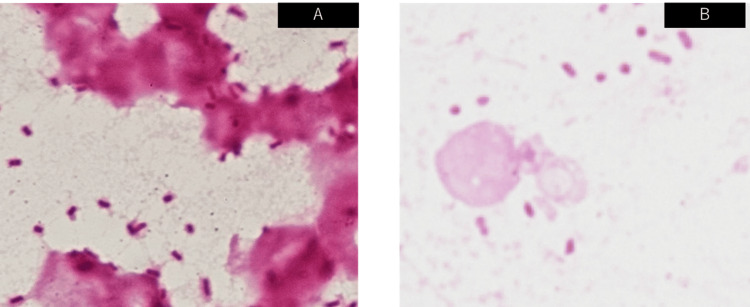
Gram stains (A) Hypermucoviscous *Klebsiella pneumoniae* was detected in blood culture. (B) A lot of gram-negative rods were found in the sputum of Geckler classification 5

**Figure 3:**
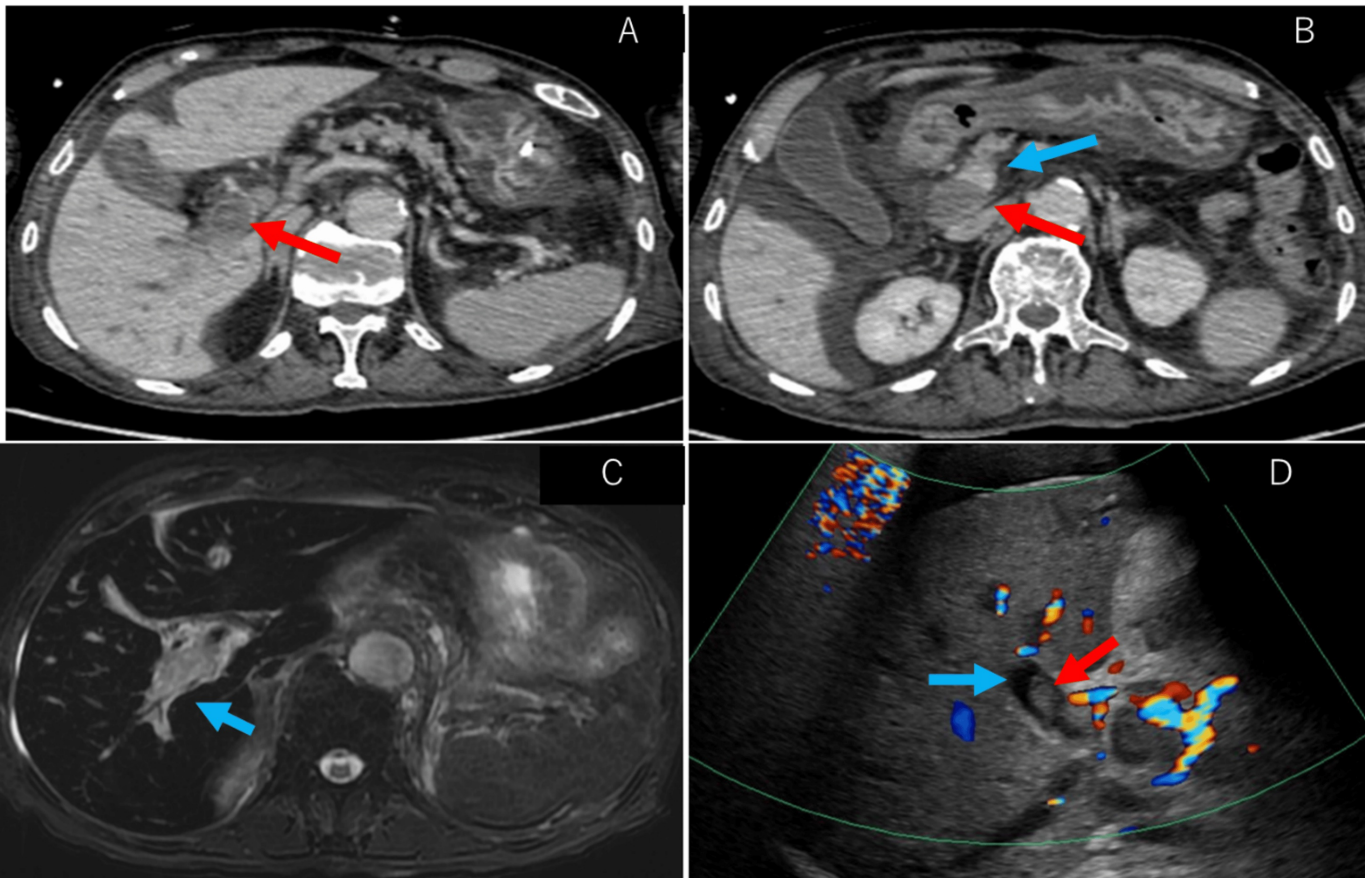
Abdominal computed tomography, magnetic resonance imaging, and ultrasonography (A and B) Contrast-enhanced computed tomography of the abdomen showed a portal vein thrombosis (indicated by the red arrow) in the main portal vein (indicated by the blue arrow). (C) The fat-suppressed T2-weighted abdominal magnetic resonance imaging scan showed a high-signal area along the portal vein, indicating the spread of inflammation. (D) Abdominal ultrasonography showed a thrombus (indicated by the red arrow) in the portal vein (indicated by the blue arrow). The flow within the portal vein had disappeared

We hypothesized that the patient was in septic and cardiogenic shock. He received fluid resuscitation, circulatory support with vasopressors, and respiratory support with oxygen therapy, which led to the stabilization of his circulatory and respiratory status. He then received digoxin and bisoprolol. His rhythm returned to sinus rhythm, and his heart rate settled around 80-100 beats per minute. Radiography and chest computed tomography (CT) revealed consolidation along the bronchi in the right upper and lower lobes, suggesting pneumonia (Figure 1A, 1B). Meropenem was initiated to treat severe pneumonia and septic shock. Blood cultures obtained on admission revealed the growth of gram-negative rod bacteria, which were string test-positive and subsequently identified as hypermucoviscous *K. pneumoniae* (Figure 2A). Similarly, *K. pneumoniae* was detected in the patient's sputum culture (Figure 2B). Since the bacterium showed susceptibility to all antibiotics except ampicillin, the patient's antibiotic regimen was lowered to ampicillin and sulbactam on day 3, and his vital signs were stabilized to a blood pressure of 138/82 mmHg and a body temperature of 37.0℃. On day 6, follow-up radiography and chest CT showed that the consolidation observed on admission had disappeared (Figure 1C, 1D). The patient's respiratory condition improved to a saturation of 96% with 2 L of oxygen, indicating that his pneumonia had improved. However, a follow-up blood culture performed on day 6 was once again positive, indicating sustained bacteremia. We conducted an abdominal contrast-enhanced CT scan to investigate the possible presence of disseminated lesions such as liver abscesses or intravascular infections. We found no signs of abscess formation in the liver, spleen, or kidneys; however, we did find portal vein thrombosis (Figure 3A, 3B). A fat-suppressed T2-weighted abdominal magnetic resonance imaging (MRI) scan showed a strong signal along the portal vein (Figure 3C), suggesting inflammation spread along the portal vein caused by an infection in the portal vein thrombosis itself. Abdominal ultrasonography revealed no blood flow in the portal vein (Figure 3D). The persistence of bacteremia despite the improvement of the patient's pneumonia, as well as the abdominal MRI findings, led us to believe that his portal vein thrombus was an infective, suppurative thrombosis rather than a pure thrombosis.

A contrast-enhanced MRI of the head did not reveal brain abscesses, and an examination by an ophthalmologist did not show signs of endophthalmitis. Based on these findings, the patient was diagnosed with pylephlebitis secondary to *K. pneumoniae* pneumonia. The *K. pneumoniae* that once again grew in the second blood culture also showed good susceptibility to antibiotics; hence, the ampicillin and sulbactam treatment was continued. From day 9 onward, apixaban was initiated as an anticoagulant therapy for portal vein thrombosis. Another blood culture on day 10 was negative, and the patient's CRP level continued to decrease. However, his bilirubin levels continued to rise, anorexia and consciousness disorders appeared, and the patient's overall condition worsened. Anemia and thrombocytopenia developed because of portal hypertension, and blood transfusion was required every day from day 20 onward. A follow-up abdominal ultrasound was performed on day 23, but there was no reduction in portal vein thrombosis, and a reverse flow of portal blood to the splenic vein was observed. Anticoagulant therapy alone was not sufficiently effective because the portal vein thrombus was too large, and invasive treatments such as thrombus retrieval using a catheter were not considered advisable because of the patient's advanced and poor overall condition. The patient ultimately died on day 27 of his hospitalization.

## Discussion

Pylephlebitis is a suppurative thrombophlebitis that occurs in the portal vein and has a reported mortality rate of 14% [1]. It typically occurs as a complication of intra-abdominal infections: primary diverticulitis (26.5%), appendicitis (22%), and liver abscess (8.5%) [1]. In our case, neither abdominal CT nor the physical examination revealed any findings suggestive of an abdominal infection. To our knowledge, this is the first report of pylephlebitis secondary to a respiratory infection. Despite the absence of diseases such as cirrhosis that cause the stagnation of portal blood flow and intra-abdominal infections, our patient's portal vein thrombosis was complicated. Several factors made our patient more prone to thrombosis. The reasons for this include the presence of MDS and DIC complications caused by both respiratory and circulatory failure. In addition, lipopolysaccharides from gram-negative bacilli damage the vascular endothelium and are involved in thrombus formation [3]. Several reports of septic pulmonary embolism caused by *K. pneumoniae* have been reported, and this bacterium may be directly involved in thrombus formation [4]. We hypothesized that these microbial factors, in addition to patient-specific factors, led to the secondary development of portal vein thrombosis as a complication of bacteremia in our patient.

Organisms that produce strings of ≥5 mm from a colony are called hypermucoviscous. Hypermucoviscous *K. pneumoniae* is thought to be resistant to phagocytosis by neutrophils, owing to its production of a capsule. It may also carry a high risk of invasive complications such as liver abscesses, meningitis, endophthalmitis, and psoas abscesses and is associated with high virulence [2]. No definitive evidence exists that hypermucoviscous *K. pneumoniae* is associated with a higher risk of thrombosis compared to non-hypermucoviscous *K. pneumoniae*. However, considering the numerous reports of liver abscesses caused by hypermucoviscous *K. pneumoniae* [5] and the fact that 30% of liver abscesses are complicated by pylephlebitis [6], high virulence has been suggested to be involved in the unusual clinical course of respiratory infections complicated by pylephlebitis.

Currently, no consensus exists on the use or duration of anticoagulation therapy for pylephlebitis. Furthermore, anticoagulant therapy contributes to the disappearance of thrombosis. For example, in a retrospective study involving 100 patients with pylephlebitis, anticoagulant therapy was associated with lower mortality [7]. The optimal choice of anticoagulant therapy remains unclear; however, heparin and direct-acting oral anticoagulants may represent promising candidates. We decided to treat our patient with apixaban, which is considered to have a lower risk of bleeding than heparin, because we anticipated the development of coagulation abnormalities and thrombocytopenia caused by his liver failure. We avoided the use of heparin because of the risk of heparin-induced thrombocytopenia.

In our case, no brain abscesses were found on contrast-enhanced MRI, and an ophthalmologist confirmed the absence of endophthalmitis. Therefore, when choosing an antibiotic, considering its ability to cross the central nervous system is not necessary. The *K. pneumoniae* detected in our patient's blood and sputum cultures was highly susceptible to all antibiotics. Therefore, several beta-lactam antibiotics have become common treatment options for this type of infection. In a retrospective study of *K. pneumoniae* liver abscesses in Taiwan, the risk of developing disseminated infection and respiratory failure was significantly higher in the group treated with cefazolin compared to the one treated with ceftriaxone [8]. Therefore, although the susceptibility was observed in vitro, cefazolin treatment was avoided. Cefmetazole can affect vitamin K metabolism and potentially cause coagulation abnormalities. Given the presence of liver failure, thrombocytopenia, and a high risk of bleeding, we decided not to administer cefmetazole to our patient. Therefore, ceftriaxone and ampicillin + sulbactam remained our primary choices. We chose ampicillin and sulbactam, which have a broader combined spectrum against anaerobes than ceftriaxone, and allow for oral switching.

This study has several limitations worth noting. First, we have not been able to directly prove portal vein thrombus infection. Persistent bacteremia and high-signal intensity on MRI along the portal vein may represent indirect evidence of thrombus infection. Second, a positive string test does not necessarily indicate a high virulence of *K. pneumoniae*. We did not examine whether the *K. pneumoniae* isolated from our patient possessed the regulator of mucoid phenotype A (*rmpA*) or mucoviscosity-associated gene A (*magA*) genes that have been associated with high virulence. Further studies on the biomarkers associated with high virulence that can be detected in general microbiology laboratories are therefore anticipated.

## Conclusions

Pylephlebitis typically occurs as a complication of intra-abdominal infection. However, we encountered a rare case of pylephlebitis complicated by pneumonia. The high virulence of *K. pneumoniae* may be involved in the formation of portal vein thrombosis. If bacteremia persists even after the pneumonia has improved, investigating the possibility of complications such as pylephlebitis is necessary.
